# Acknowledgment to Reviewers of *Veterinary Sciences* in 2020

**DOI:** 10.3390/vetsci8020018

**Published:** 2021-01-25

**Authors:** Veterinary Sciences Editorial Office

**Affiliations:** MDPI AG, St. Alban-Anlage 66, 4052 Basel, Switzerland

Peer review is the driving force of journal development, and reviewers are gatekeepers who ensure that *Veterinary Sciences* maintains its standards for the high quality of its published papers. Thanks to the cooperation of our reviewers, in 2020, the median time to first decision was 20 days and the median time to publication was 38 days. The editors would like to express their sincere gratitude to the following reviewers for their precious time and dedication, regardless of whether the papers were finally published:
Abd El-Aziz, Tarek MohamedLeinonen, HenriAbdelhakiem, Mohammed A. H.Leitão, AlexandreAbdellatif, AhmedLeite, Fábio Pereira LeivasAbidu-Figueiredo, MarceloLeonardi, FabioAbramo, FrancescaLeroux, Louis-PhilippeAcharya, Kamal RajLestingi, AntoniaAdamiak, ZbigniewLewkowski, OlegAguiar, Daniel M.Li, AnningAlagwany, MahmoudLi, JilianAlbertini, MariangelaLiao, Albert T.Alcalde, Maria J.Liao, Ling-HsiuAlegre, BeatrixLiatis, TheophanesAleri, JoshuaLin, Chen-SiAlexander, Brenda M.Ljubimov, AlexanderAlibhai, Hatim I. K.Loi, FedericaAl-Sagheer, AdhamLøvdal, TrondAlves, Carlos RobertoLu, Jeng-WeiAmemiya, KeiLücke, Friedrich-KarlAmiri, EsmaeilLühken, RenkeAndré, Marcos RogérioLulich, JodyAndré-Fontaine, GenevieveLux, Cassie N.Andreo, VeronicaLyons, Leslie A.Anglani, FrancaMacrì, FrancescoAngrand, Pierre-OlivierMadoroba, EvelynAranaz, AliciaMagalhães Sant′Ana, ManuelArmson, BryonyMagallanes, SergioAruguete, DeborahMainil, JacquesAttia, Youssef A.Manfredi, Maria TeresaAugustine, Swinburne A. J.Maniaci, GiuseppeAugustyniak, AdrianManino, AuloAuvray, FrédéricMansell, PeterAvalos, ArianMarks, Stanley L.Awosile, BabafelaMaros, KatalinAzam, Aa HaerumanMarsilio, FulvioBajcsy, ÁrpádMartin, StephenBalasubramanian, BalamuralikrishnanMartini, ValeriaBannantine, John P.Martinković, FranjoBao, WenbinMartino, Luisa DeBarański, WojciechMatera, Julia MariaBarbet, AnthonyMateus, Luísa MariaBarbosa, AngelaMatos, Augusto J. F.Barbosa, BrunoMatsuda, AkiraBarjesteh, NedaMatte, Alisha R.Barrows, MichelleMcAuley, Catherine M.Bartosik, KatarzynaMcGiven, JohnBąska, PiotrMcGuire, BettyBatista Sampaio, CláudiaMeeker, Rick B.Bazzano, MarilenaMelchiorre, DanielaBeard, PipMellado, MiguelBeaurepaire, AlexisMellies, JayBelbis, GuillaumeMendonça, TiagoBellezza, EnricoMenozzi, AlessandroBellini, SilviaMerino, Eva Maria PerezBełżecki, GrzegorzMichnik, Robert A.Bender, Scott C.Miglio, AriannaBenítez, Hugo A.Minervino, Antonio Humberto HamadBertelloni, FabrizioMitchell, Scott G.Bertzbach, Luca DaniloMomotani, EiichiBeths, ThierryMontoya Alonso, Jose AlbertoBhat, SushantMorelli, SimoneBlanco, EstherMorfin, NuriaBlanton, CynthiaMunday, JohnBlome, SandraMusallam, ImadiddenBoehm, DanielaMusella, VincenzoBoelke, MathiasMusk, Gabrielle C.Bonelli, FrancescaMutinelli, FrancoBonelli, PieroMuttini, AurelioBonnet, Alejandro SuarezNagy, BelaBonsembiante, FedericoNahashon, Samuel N.Boone, JohnNatale, AldaBoraschi, DianaNathanailides, CosmasBordes, María Dolores LlobatNavas, LuigiBorriello, GiorgiaNazki, SalikBorsuk, GrzegorzNeustupa, JiriBouhsira, EmilieNewcomer, Benjamin W.Bovera, FulviaNguyen, FrédériqueBrachelente, ChiaraNguyen, ThuBragg, RobertNiedringhaus, Kevin D.Brown, Bryan R.Nissapatorn, VeeranootBrown, Mark A.Nixon, JaneBrutscher, LauraNizza, AntoninoBuonavoglia, DomenicoNkukwana, ThobelaBurges, GrahamNomoto, RyoheiBurrai, Giovanni PietroNunes, Francis Morais FrancoBurritt, James B.Occi, JamesBustamante, Hedie A.Odemer, RichardBustos-Martínez, JaimeOgrzewalska, MariaByrd, Christopher J.Ohmori, KeitaroByrne, AlexanderOhta, MitsuakiCabirol, AmélieOliva, GaetanoCain, Christine L.Oliveira, ManuelaCala, StevenOliver, JamesCaldas, Lucio AyresOlsen, RikkeCameron, LornaOrtalda, ChristianCampler, Magnus R.Ortuño, AnnaCândido Carvalho, KátiaOrusa, RiccardoCarcangiu, VincenzoOzella, LauraCareem, FaizalPaixão, Tatiane AlvesCarney, Hazel C.Palma Sircili, MarceloCastel, AudePang, LisaCazapal-Monteiro, Cristiana FilipaPaoletti, BarbaraCenac, NicolasPapadopoulos, GeorgiosCerquetella, MatteoParambeth, Joseph CyrusChai, JinParés-Casanova, Pere M.Chase, LarryParisi, FrancescaChaves, Luis FernandoParnell, Laura K. S.Chen, CortinaPasquale, Jorgelina DiChen, Yi-NingPassantino, AnnamariaChitimia-Dobler, LidiaPassero, FelipeCho, JeongheePatra, Amlan KumarChowdhury, Abu SayedPaudyal, SushilChristensen, HenrikPavarini, Saulo PetinattiCiaramella, PaoloPaz-Silva, AdolfoCilia, GiovanniPenning, LouisCiliberti, Maria GiovannaPenrith, Mary-LouiseCiti, SimonettaPepi, MilvaClegg, SimonPerego, RobertaClemente, LurdesPérez-Martínez, ClaudiaCodd, Jonathan R.Pérez-Pascual, DavidColumbano, NicoloPeriago, Maria VictoriaComazzi, StefanoPerrucci, StefaniaConstantino-Casas, FernandoPesavento, PatriciaCooney, KathleenPeterson, Karianne LievaartCooper, KimberlyPiantedosi, DiegoCorda, AndreaPiantino Ferreira, Antonio JoséCork, SusanPickett, AndyCorpas-López, VictorianoPickup, RogerCorrente, MarialauraPienaar, RonelCotovio, MárioPierini, AlessioCouetil, Laurent L.Pieszka, MarekCrupi, RosaliaPires, GraçaCuevas-Ramos, GabrielPirk, Christian W. W.Czopowicz, MichałPisoni, LucianoDa Luz E Silva, SauloPisu, Maria CarmelaDalla Villa, PaoloPohlmann, AnneDamiaans, BertPoli, AlessandroDance, David A. B.Porcellato, IlariaDaniels, SimonPorporato, MarcoDas, UndurtiProvot, ThomasD'Ascenzi, CarloPtacek, MartinDavid, González-BarrioPuff, ChristinaDawood, Mahmoud A. O.Puggioni, AntonellaDe Briyne, NancyQueiroz, Luzia HelenaDe Carvalho, Gleidson Giordano PintoQurollo, BarbaraDe Cramer, Kurt G. M.Rajamohan, Arunde Graaf, DirkRajeev, SreekumariDe Jesus, CarrieRambozzi, LuisaDe Rooster, Hilde H.Raskovic, BozidarDe Simone, Salvatore GiovanniRattanapan, CheerawitDec, MartaRauser, PetrDeisig, NinaRavera, IvanDel Chicca, FrancescaRégnier, AlainDellamaria, DeboraRego, RyanDell'olmo, ElianaReichel, Michael P.Deng, XiaolanReid, ScottDevriendt, BertReifinger, MartinDevriendt, NausikaaReiko, NagataDewals, BenjaminReines, Brandon P.Di Francesco, Cristina EsmeraldaReis, AnaDias-Pereira, PatriciaRen, LinzhuDiederich, SandraRenz, AlfonsDimitrov, Kiril M.Reynaldi, Francisco JoséDing, LumingRichards, Allen L.Dixon, LindaRieder, ElizabethDjokovic, RadojicaRigano, DanielaDmitryjuk, MałgorzataRizk, Mohamed AbdoDo, Duy NgocRoberts, JohnDodds, W. JeanRodrigo Mocholi, DiegoDolan, Emily D.Rodriguez, CarlosDolgova, OlgaRodriguez, NildaDomingo-Calap, PilarRohaim, Mohammed A.Domínguez Rebolledo, Álvaro EfrénRomar, RaquelDomínguez-Bernal, GustavoRoncada, PaolaDomínguez-Pérez, DanyRönnberg, HenrikDorny, PierreRose, NicolasDos Anjos Pires, MariaRossi, LucaDos Santos Sousa, Carlos AugustoRoura, XavierDos Santos, Regiane RodriguesRowan, AndrewDowns, KevinRyabov, EugeneDrake, Adryanna SiqueiraSaadeldin, IslamDubovi, EdwardSakai, HirokiDyson, SueSakamoto, Joyce M.Dzimira, StanisławSaki, NajmaldinEbensperger, GermánSammi, ShreeshEdamura, KazuyaSamuel, RyanEdwards, Gaylen L.Santifort, Koen M.Edwards, John F.Savatier, PierreEgelund, Eric FreeSavvas, IoannisEkateriniadou, LoukiaScandurra, AnnaEl-Khadragy, Manal F.Schäfer-Somi, SabineElleder, DanielScharf, ValeryElmi, AlbertoSchusser, Gerald FritzElolimy, AhmedSchwarz, TobiasElrod, Susan M.Schwermer, HeinzpeterElsayed, Ahmed MostafaScocco, PaolaElshafie, Hazem S.Scofield, RoseEl-Sheikh Ali, HossamSegundo, MarcelaErickson, PeterSeijo, M. CarmenEschbaumer, MichaelSevigny, MaryFair, JeanneSevilla-Morán, BeatrizFarnworth, Mark J.Sgorbini, MicaelaFasina, Folorunso OludayoShakeri, MajidFeás, XesúsShao, HuabinFederica, PirroneSharafeldin, TamerFei, Andrew Chang-YoungSharma, ArvindFelicioli, AntonioShekhova, ElenaFelix, AnandaShemilt, SueFerraz, PatriciaShi, Xin’eFerro, SilviaShimizu, MikiFinotello, RiccardoSilva, LuizFlisikowski, KrzysztofSilvestrini, PaoloFloris, RosannaSingh, ShailbalaFonseca-Alves, Carlos EduardoŠiroký, PavelForget, PatriceSkaldina, OksanaForlani, AnnalisaSmith, Heather F.Fortina, RiccardoSolaiman, Sandra G.Francisco, Alexandra E.Soler, MartaFratini, FilippoSolovyev, NikolayFrean, JohnSomasundaram, Dinesh BabuFregonesi, José AntonioSøvik, EirikFrossard, Jean-PierreSpada, EvaFthenakis, GeorgeSpadari, AlessandroFusellier, MarionSpadavecchia, ClaudiaGabler, ChristophSpadola, FilippoGabriel, AuraSpina, AlfioGajda, AnnaSplichalova, AllaGalisteo Jr., Andres JimenezStaffieri, FrancescoGallo, CarmenStancampiano, LauraGanz, Holly H.Stanford, KimGarcía-Rebollar, PalomaStanimirovic, ZoranGavazza, AlessandraStannard, Wild Hayley J.Geiger, MadeleineSteinman, AmirGiacomini, PatrizioSter, CélineGiacomo, GnudiSternberg-Lewerin, SusannaGiannenas, IliasStreck, André FelipeGilbert, AmyStromberg, ZacharyGinaldi, LiaSun, LvhuiGoblirsch, Michael J.Suzuki, RyoheiGoethem, Bart VanSzentgyörgyi, HajnalkaGonzalez Montana, Jose RamiroSzulczyński, BartoszGonzález-Martín, Juan V.Szweda, PiotrGonzález-Ríos, HumbertoTaddei, SimoneGoodla, LavanyaTakehiro, NishidaGorska-Ponikowska, MagdalenaTallo-Parra, OriolGould, Ernest A.Tambella, Adolfo MariaGoyal, SagarTanui, Collins K.Goździewska-Harłajczuk, KarolinaTarantola, MartinaGraham, John H.Tassis, PanagiotisGreenhalgh, DavidThamsborg, Stig MilanGrieco, ValeriaThangathurai, DuraiyahGuérios, Simone DomitThomas, Donald B.Gunesekera, AmilaTokarska, MałgorzataGupta, KuldeepTokarz, Debra A.Gutierrez-Blanco, EduardoTrevisani, MarcelloHahn, CarolineTroan, BrigidHall, EdwardTsai, Kun-HsienHällgren, AnitaTsang, Chi-ChingHam, KathleenTsiouris, VasiliosHanau, StefaniaTsironi, FannyHanlon, Alison J.Tufarelli, VincenzoHariharan, HarryTurner, Patricia V.Hassler, EdgarTurni, ConnyHauser, PeterTydén, EvaHäußler, SusanneUlicna, LiviaHayssen, VirginiaUlrich, ReinerHeadley, Selwyn ArlingtonUmemura, AkiraHellmen, EvaUnderwood, RobynHenao-Díaz, Yuly AlexandraUyeno, YutakaHendriksma, HarmenVan Der Steen, Jozef J. M.Hidalgo, HéctorVan Doorn, Deborah C. K.Hildreth, Blake E.Van Wettere, ArnaudHill, Richard C.Vasicek, OndrejHisasue, MasaharuVasileiou, Natalia G. C.Hofmeister, ErikVecchiato, Carla GiudittaHuntingford, JaniceViboud, GloriaHussein, MaythamViegas, CarlosIjichi, CarrieVignoli, MassimoImai, TakashiVilar Ares, Maria J.Inoue, SatoshiVillalobos, Ethel M.Isoda, NorikazuVillarruel-López, AngélicaItzkowitz, MurrayVilte, Daniel A.Iwasaki, JuaVolta, AntonellaIzdebska, Joanna N.Vos, AdJahns, HanneVrentas, Catherine E.James, JoeWaal, Theo DeJamil, TariqWalker, DavidJanowicz, AnnaWalsh, Catherine J.Jansson, DésiréeWalton, Stuart A.Jia, BeibeiWang, FangJødal, LarsWang, Jiu-FengJung, Dong-InWang, Sheng-yiKalaitzakis, EmmanouilWang, Shu-YinKamdem, Jean PaulWareth, GamalKan, Chi-waiWarrell, MaryKanda, TeppeiWashburn, SteveKato, AtsushiWasniewski, MarineKatsoulos, Panagiotis D.Waterhouse, TonyKaur, HarleenWebster, Thomas C.Kawasaki, KiyonoriWennogle, Sara A.Keenan, Jacqueline I.Weston, Jennifer F.Kezic, NikolaWestreicher-Kristen, EdwinKim, Jong-HyukWhent, MonicaKim, Ki-YoungWhitaker, JustineKnight, PeterWhittaker, AlexandraKock, RichardWilde, JerzyKogut, MichaelWolf, PeterKokoszyński, DariuszWoltenberg, LeslieKoltes, DawnWood, Sarah C.Komlósi, IstvánWu, Jack H. Z.Kong, XiangfengXu, XiaoKowalik, Magdalena K.Xu, XuewenKrause, Alexandre J.Yadav, DhananjayKrömker, VolkerYamamoto, TakeshiKryger, PerYamazaki, TaigaKulkarni, RaviYang, Vicky K.Kupczyński, RobertYe, JianqiangKusano, KanichiYonekura, ShinichiKyriakis, Constantinos S.Yzydorczyk, CatherineLacitignola, LucaZecconi, AlfonsoLafontant, Pascal J.Zerbe, HolmLamagna, BarbaraZgank, AndrejLambertini, CarlottaZhang, GuanshiLang, AndrewZhang, JileiLanteri, GiovanniZhang, KaiLappalainen, Anu K.Zhang, ShiyongLaven, LindaZheng, AijuanLaven, RichardZhou, ShuanhuLe Gall, TonyZhu, LiqianLeal, Rodolfo OliveiraZowalaty, Mohamed E. ElLederman, ZoharZsolnai, AttilaLee, Jong-HaZucca, EnricaLee, Tzu-TaiZwierzchowski, GrzegorzLéguillette, Renaud


